# Isotemporal substitution of sedentary time with physical activity for cardiovascular health in older adults: a systematic review

**DOI:** 10.3389/fspor.2026.1708003

**Published:** 2026-02-25

**Authors:** Xu Sun, Zongkai Zhou, Junyi Guo, Zuguo Tian, Nina Gu

**Affiliations:** Hunan University, Changsha, China

**Keywords:** cardiovascular health, isochronic substitution models, older adults, physical activity, sedentary behavior

## Abstract

**Background:**

Cardiovascular disease (CVD) is the leading cause of death among older adults, with sedentary behavior (SB) as a key modifiable risk factor. While physical activity (PA) is associated with cardiovascular health, evidence remains limited on the specific effects of replacing SB with PA of varying intensities.

**Objective:**

To systematically review evidence on the cardiovascular effects of substituting SB with PA in adults aged 65 and older using isotemporal substitution modeling (ISM).

**Methods:**

Following PRISMA guidelines, seven databases were searched up to April 2025. Risk of bias was assessed using the JBI tool, and a narrative synthesis was conducted.

**Results:**

Eighteen observational studies (15 cross-sectional, 3 cohorts) using ISM were included. Replacing 10–60 min of SB with moderate-to-vigorous PA (MVPA) was associated with more favorable in blood pressure, triglycerides, waist circumference, inflammatory markers (CRP, IL−6, GDF−15), and insulin sensitivity (HOMA-IS, Matsuda-ISI). Light-intensity PA (LPA) showed modest associations, particularly among frail or mobility-limited individuals. A daily substitution of 30 min was identified as a feasible reference window, with ≥60 min linked to additional vascular and autonomic benefits.

**Conclusions:**

Replacing SB with PA, especially MVPA, was consistently associated with favorable cardiovascular profiles in older adults. Even brief substitutionsmay be beneficial, supporting intensity-stratified public health strategies and refinement of physical activity guidelines for aging populations.Because most included studies were cross-sectional, these findings should be interpreted as associations rather than definitive causal effects, and reverse causation remains a plausible concern.

**Systematic Review Registration:**

https://www.crd.york.ac.uk/prospero/view/CRD420251021829/1/0, PROSPERO CRD420251021829.

## 1 Introduction

Population ageing is accelerating worldwide (1), raising urgent public health challenges related to chronic disease prevention. Cardiovascular disease (CVD) remains the leading cause of death globally, and the expanding older population is now a primary driver of the growing CVD burden (2). In the United States, for example, adults aged ≥65 account for over 80% of all CVD-related deaths (3), reflecting the steep age-related increase in cardiovascular risk. Sedentary lifestyles compound this burden: insufficient physical activity and excessive sedentary behavior are well-established contributors to cardiovascular risk (4). Notably, total sedentary time tends to rise with age (5), as many older adults spend a large portion of their day in inactive pursuits (e.g., prolonged sitting and television viewing). This confluence of an ageing demographic and high prevalence of physical inactivity underscores the need for effective strategies to safeguard cardiovascular health in older adults.

One emerging approach to understanding and improving lifestyle risk factors is the isotemporal substitution model (ISM) in epidemiology (6). The ISM is a robust analytical framework designed to evaluate the potential health effects resulting from reallocating a fixed amount of time from one specific behavior to another, while keeping the time spent on all remaining activities constant. By modeling these time reallocations within the finite 24-h day, this approach allows researchers to isolate and quantify the relative health impact of different behavioral trade-offs, offering a more ecologically valid representation of real-world time use and its implications for public health (7). Because daily time is finite, increasing time spent in a beneficial activity (such as exercise) inherently requires decreasing time spent in another (such as sedentary behavior); ISM allows researchers to quantify the effect of “trading” one behavior for another while holding total time constant (8). Using this approach, a growing body of research has explored how replacing sedentary time with physical activity might improve health outcomes (9, 10). A 2018 scoping review of 56 studies found that reallocating sedentary time to either light physical activity (LPA) or moderate-to-vigorous physical activity (MVPA) was generally associated with favorable health indicators, including lower mortality and better cardiometabolic profiles, with larger benefits observed for substitutions involving higher-intensity activity (4). Consistently, recent prospective studies with device-based measurements report that high sedentary time is linked to elevated CVD risk, whereas greater physical activity, even at light intensity, confers significant risk reduction (11). For example, an ISM-based analysis in a large cohort (UK Biobank) estimated that replacing 1 h per day of sitting with an equal amount of physical activity was associated with a 6%–23% lower incidence of coronary heart disease, depending on the type and intensity of activity with the greatest benefit seen for vigorous exercise (12). Such findings reinforce long-standing epidemiological evidence that “moving more and sitting less” can substantially improve cardiovascular health outcomes in adult populations.

Despite these advances, important knowledge gaps and inconsistencies remain in the current evidence base. Remarkably, prior reviews have highlighted a dearth of studies focusing specifically on older adults (4), who may have different activity patterns, capacities, and risk profiles compared to middle-aged populations (13). Only in recent years have analyses begun to apply the ISM in older cohorts—examining outcomes such as functional capacity and frailty—yet the findings have not always been consistent (14). In particular, the cardiovascular and health benefits of replacing sedentary time with lower-intensity activity in older adults remain uncertain (15, 16). Some studies suggest that even light-intensity activity can yield meaningful improvements—for instance, one 15-year longitudinal study reported that substituting sedentary behavior with light activity reduced both all-cause and CVD mortality in older individuals (5). However, other evidence indicates that the effects of light activity may be limited or context-dependent in this age group (17–20). Discrepancies in findings may stem from heterogeneity in study designs (cross-sectional vs. longitudinal), differences in how physical activity was measured (objective accelerometry vs. self-report), and potential biases such as reverse causation, whereby underlying poor health leads to more sedentariness in observational studies of older adults. Furthermore, many studies did not capture the full 24-h activity cycle, for example, failing to account for sleep time, which is a significant limitation when assessing time reallocations (4). To date, no systematic review has synthesized the evidence on sedentary time substitution specifically for older adult populations, making it difficult to draw firm conclusions or craft age-tailored recommendations. This gap highlights the need for a focused appraisal of the literature to determine how and to what extent reallocating sedentary time to physical activity can benefit the cardiovascular health of older adults.

The present systematic review seeks to provide a timely and focused synthesis of the emerging evidence on sedentary behavior substitution and cardiovascular health in older adults. This undertaking is warranted by three interrelated developments. First, sedentary behavior has gained recognition as an independent risk factor for chronic disease, prompting the World Health Organization (2020) to recommend that older adults reduce sedentary time by replacing it with physical activity of any intensity (21). Second, a growing number of recent studies—especially large-scale cohort analyses—have generated new data specific to older adults that were not incorporated in earlier reviews (22). These studies suggest that even modest reallocations of sedentary time (e.g., 30 min per day) to light-intensity physical activity may be associated with differences in frailty, physical function, and metabolic profiles. Third, methodological advances such as accelerometer-based behavioral monitoring and isotemporal substitution modeling (ISM) (23) now allow for a more precise examination of time-use reallocation and its health consequences in geriatric populations. Taken together, these developments create an opportunity—and a need—for a targeted, methodologically robust review. Unlike previous studies that have primarily focused on younger populations—such as preschool children, university students, or middle-aged adults (24–26), this review is tailored to adults aged ≥65 years, whose activity patterns, functional constraints, and cardiovascular risk profiles may make time-reallocation evidence particularly sensitive to study design and intensity classification. We, therefore, aim to consolidate and appraise the current observational evidence in this age group, while explicitly acknowledging key sources of uncertainty (e.g., cross-sectional designs, reverse causation, and heterogeneity in intensity definitions and ISM specifications). By organizing findings by substitution duration and intensity (LPA vs. MVPA), we seek to clarify where associations appear comparatively consistent and where they remain context-dependent, thereby providing a practical reference for future longitudinal research and for cautious guideline discussions in ageing societies.

## 2 Materials and methods

The review methodology is designed in accordance with the PRISMA 2020 guidelines for systematic reviews (27). A detailed protocol (including search strategy, inclusion criteria, and analysis plan) is registered with the International Prospective Register of Systematic Reviews (PROSPERO) (Registration ID: CRD420251021829). We implemented a five-step approach aimed at identifying key themes and synthesizing relevant evidence (28, 29).

### 2.1 Define the research questions (RQs)

This systematic review aims to answer the following key questions regarding the isotemporal substitution of sedentary time with physical activity in older adults:

RQ1: What is the association between replacing sedentary time with physical activity and cardiovascular health outcomes in older adults?

RQ2: Does the health impact of substituting sedentary time depend on the intensity of physical activity (e.g., light vs. moderate-to-vigorous)?

### 2.2 Search strategy

A comprehensive literature search was conducted between 5 and 13 April 2025 across the following sources: Web of Science Core Collection, PubMed, Embase, Scopus, EBSCOhost, ProQuest, and Wiley Online Library. Searches were run using the same Boolean query across all sources to ensure conceptual consistency and to facilitate reproducibility. Platform-specific adjustments were limited to syntax required by each interface (e.g., quotation marks and truncation symbols), without altering the underlying search concepts. For EBSCOhost and ProQuest, searches were performed via the platforms’ Advanced Search interfaces with the search scope set to all databases available through the authors’ institutional subscriptions at the time of searching. For Wiley Online Library, searches were conducted using the Advanced Search function across journal content. The final search string used in this review was: (“older adults” OR “elderly” OR “aged” OR “geriatric population” OR “retirees” OR “65 + years” OR “aged 65 years and older”) AND (“physical activity” OR exercise OR “sports participation” OR “walking” OR “jogging” OR “tai chi” OR “yoga” OR “household chores” OR “moderate-intensity activity” OR “vigorous-intensity activity” OR METs) AND (“sedentary behavio*” OR “sedentary time” OR “sitting time” OR “TV viewing” OR “prolonged sitting” OR “screen time” OR “occupational sitting”) AND (“isotemporal substitution” OR “time substitution model” OR “activity replacement” OR “replace sedentary time with exercise” OR “24-h activity reallocation” OR “behavioral displacement”) AND (“cardiovascular health” OR “cardiovascular disease*” OR “cardio-metabolic health” OR “blood pressure” OR “HDL” OR “LDL” OR “triglycerides” OR “coronary heart disease” OR “stroke”).

There are no restrictions on the publication start date for the search. The search results were last updated on April 13, 2025. Inclusion criteria are limited to English-language literature with human subjects (as supported by the database interface). During screening, strict adherence was maintained to inclusion criteria regarding elderly participants (typically ≥65 years) and peer-reviewed full-text availability. Additionally, reference lists of included studies and relevant reviews were manually searched to identify other eligible studies.

### 2.3 Inclusion and exclusion criteria

Eligible studies were included in this systematic review if they fulfilled the following criteria: (a) peer-reviewed original research focused on older adults, typically defined as individuals aged 65 years and above; (b) investigations reporting at least one cardiovascular health-related outcome, such as systolic or diastolic blood pressure, resting heart rate, incidence of cardiovascular disease, or cardiometabolic markers (e.g., lipid profiles, glucose regulation, inflammatory biomarkers); (c) studies utilizing isotemporal substitution modeling (ISM) to assess the hypothetical effects of reallocating time from sedentary behaviors—such as total sedentary time, sitting duration, or sedentary patterning—toward various domains or intensities of physical activity, including light (LPA), moderate (MPA), vigorous (VPA), or combined moderate-to-vigorous physical activity (MVPA), as well as standing postures; and (d) publications written in English.

Studies were excluded if they met any of the following conditions: (a) interventional or experimental trials, including randomized controlled trials (RCTs), which do not align with the observational nature of ISM-based analyses; (b) studies not employing an isotemporal analytical framework; (c) articles lacking explicit cardiovascular outcome measures; or (d) reviews, conference abstracts, editorials, commentaries, dissertations, or other non–peer-reviewed literature.

### 2.4 Study selection and data extraction

At the outset, one reviewer (Sun) conducted the database searches and imported all retrieved records into EndNote X9 (Thomson Research Soft) for systematic deduplication, and a second reviewer (Guo) checked the search records and deduplication outputs.Then, the study selection process was conducted in two sequential phases to ensure methodological rigor. In the initial phase, two reviewers (Sun and Guo) independently screened the titles and abstracts of all retrieved articles based on the predefined inclusion criteria (outlined in Table 1). Articles that clearly did not meet the eligibility requirements were excluded at this stage. In the second phase, the full texts of potentially relevant studies were retrieved and independently assessed by the same two reviewers (Sun and Guo). The eligibility of each study was evaluated using the same inclusion and exclusion criteria applied during the initial screening. Any discrepancies or disagreements encountered during either phase were resolved through discussion, and if consensus could not be reached, a third reviewer (Tian) was consulted to arbitrate the decision. The entire selection process, including the number of studies screened, excluded, and included, along with reasons for full-text exclusions, was documented in accordance with PRISMA guidelines and is illustrated in the PRISMA flow diagram (27).

**Table 1:** PICOS included in the study.

Category	Inclusion criteria	Exclusion criteria
Population	Older adults (typically ≥65 years of age) in study populations.	Youth or general adult populations without a specific older adult focus.
	Human participants only.	Non-human studies.
Interventions	Sedentary behavior and physical activity time use assessed via accelerometry or validated self-report/recall instruments.	Studies not employing time reallocation analysis (no isotemporal).
	Analysis uses isotemporal substitution or compositional data models.	Interventional studies manipulating behavior (e.g., exercise trials).
Comparators	Implicit comparisons of time spent in different activity behaviors via reallocation (e.g., replacing sedentary time with equal LPA or MVPA).	No comparison of activity reallocation (e.g., single-behavior models with no substitution analysis).
Outcomes	Cardiovascular health outcomes (e.g., blood pressure, cardiovascular disease incidence, resting heart rate).	Outcomes not related to cardiovascular health (e.g., cognitive, mental health, musculoskeletal outcomes).
	Cardiometabolic biomarkers (e.g., cholesterol, glucose levels).	
Study design	Observational studies (cross-sectional surveys, longitudinal cohort studies).	Randomized or non-randomized trials or other intervention studies.
	English-language, peer-reviewed full text available.	Non-English publications: reviews, editorials, or protocol-only papers.

A standardized data extraction form was developed and piloted to ensure consistency across studies. Two reviewers (Guo and Zhou) independently extracted data, with discrepancies resolved through discussion and verification against the original articles. Extracted variables included study characteristics (author, year, country, design, sample size, and participant age), definitions and assessment methods of sedentary behavior and physical activity, analytical approaches including use of isotemporal substitution, comparator conditions (e.g., behaviors substituted and duration of reallocation), and covariates adjusted for in the models. Key cardiovascular health outcomes and effect estimates were extracted, including the direction and magnitude of associations for each modeled time reallocation, such as the impact of replacing sedentary time with light or moderate-to-vigorous physical activity on blood pressure, cardiovascular events, or biomarker profiles.

### 2.5 Quality evaluation and data analysis

To appraise the methodological quality and assess the risk of bias of the included observational studies, we employed the Joanna Briggs Institute (JBI) Critical Appraisal Checklists for Analytical Cross-Sectional Studies and for Cohort Studies, both specifically designed to evaluate non-randomized epidemiological research (30). The JBI tool for cross-sectional studies encompasses eight domains, and the cohort study checklist expands this assessment to eleven domains, additionally incorporating key longitudinal elements such as confirmation of baseline outcome-free status, adequacy and completeness of follow-up, and appropriate handling of attrition (31). Each included study was independently appraised by two reviewers (Sun and Guo), with discrepancies resolved through consensus discussion; if disagreement persisted, a third reviewer (Zhou) served as the final arbiter. For quality classification, cross-sectional studies scoring 7–8 were deemed high quality (low risk of bias), 5–6 moderate quality (moderate risk of bias), and ≤4 low quality (high risk of bias). Similarly, cohort studies scoring 9–11 were categorized as high quality, 6–8 as moderate quality, and ≤5 as low quality. This standardized appraisal framework ensured a systematic and transparent evaluation of study rigor. Given anticipated heterogeneity across studies in terms of design (e.g., cross-sectional vs. longitudinal), measurement methods (device-based vs. self-report), and outcome reporting (e.g., blood pressure, cardiovascular events, biomarker profiles), we judged quantitative pooling inappropriate. Additional heterogeneity was expected in the operationalization of activity intensity (e.g., ENMO- or count-based cut-points vs. MET-derived thresholds, as well as differences in device placement and processing algorithms) and in ISM specifications [standard ISM vs. compositional approaches using isometric log-ratio (ILR) transformation]. Such variability limits the direct comparability of effect estimates across studies and is particularly relevant for LPA, where classification boundaries are more sensitive to threshold choice.Therefore, a structured narrative synthesis was undertaken to interpret the findings in light of study quality and methodological diversity (32).

## 3 Results

### 3.1 Study selection

**Figure 1:**
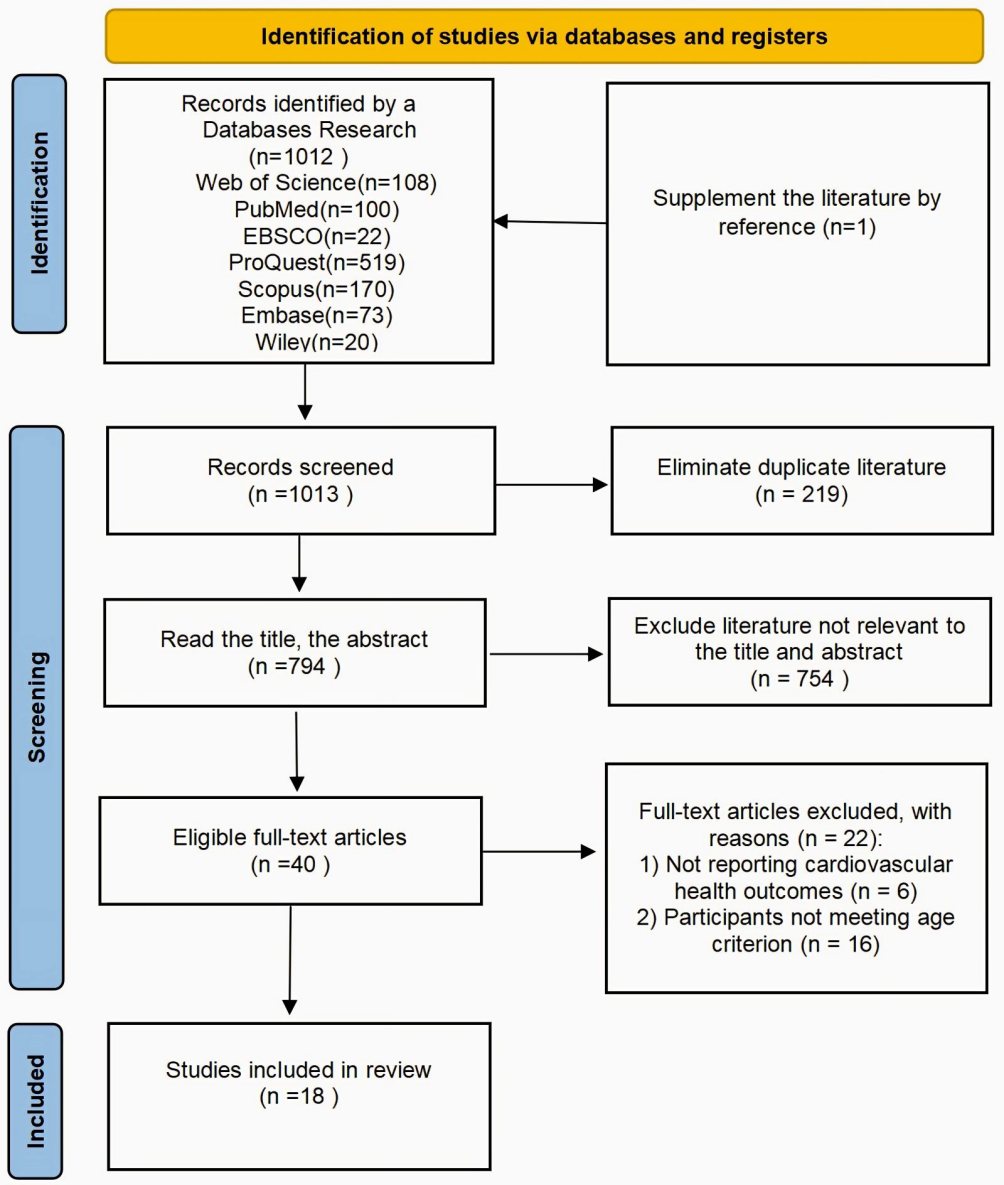
Literature screening flowchart.

A total of 1,012 publications were obtained through database searches in this study from the following sources: Web of Science (*n* = 108), PubMed (*n* = 100), EBSCO (*n* = 22), ProQuest (*n* = 519), Scopus (*n* = 170), Embase (*n* = 73), and Wiley (*n* = 20). One piece of literature was obtained through reference supplementation, giving an initial total of 1,013 pieces of literature. On this basis, all the literature was imported into EndNote X9 software for deduplication, and 219 duplicate records were removed, leaving 794 unique records. Then, two reviewers (Sun and Guo) independently screened the titles and abstracts of the 794 papers according to the predefined eligibility criteria, and excluded 754 papers that were not relevant to the topic and/or did not meet the inclusion criteria. A total of 40 papers were entered into the full-text reading stage, and two reviewers (Sun and Guo) independently read the full text to assess eligibility, excluding 22 full-text articles with reasons. Specifically, 6 articles did not report cardiovascular health-related outcomes, and 16 studies did not meet the age criterion for older adults (≥65 years). Any discrepancies at either stage were resolved through discussion, and if consensus could not be reached, a third reviewer (Tian) was consulted to arbitrate the decision. Ultimately, 18 studies were included in the review. The literature screening process is detailed in Figure 1.

### 3.2 Research characteristics

The 18 studies included in this study, spanning the period from 2014 to 2024, were conducted in the UK (*n* = 8) (16, 33–39), the United States (*n* = 4) (40–43), Australia (*n* = 2) (10, 14), Sweden (*n* = 2) (44, 45), Canada (*n* = 1) (46), and Spain (*n* = 1) (47), with a total of 15 cross-sectional studies and 3 prospective cohort studies. Specifically, (1) The studies were all conducted in the elderly population, with a mean age of 65 years and older, and sample sizes ranging from 54 to 16,031 cases, with four studies explicitly limited to the female elderly population, reflecting the population specificity of some of the studies (2). In terms of measurement tools, most included studies adopted device-based measures to quantify physical activity and sedentary behaviour, using monitors such as ActiGraph GT3X, GENEActiv, and activPAL, with common wear locations including the hip, thigh, and wrist, and only one study used a subjective recall method, namely the NIH-AARP Diet and Health Study Questionnaire (3). In terms of modeling methods, all studies used isotemporal substitution modeling (ISM). Thirteen of the studies used standard ISM modeling under traditional linear regression, and another five used combined or compositional ISM, which was mainly controlled by ILR transformation (isometric log-ratio transformation) with multivariate covariance structure, reflecting the evolution of the complexity of modeling the relationship of behavioral combinations. This reflects the evolving complexity in modeling behavioral combinations, indicating a shift toward more composition-aware modeling approaches that use ILR transformation to handle the constrained 24-h time composition and estimate reallocation effects within a closed time budget (4). Regarding the classification of activity intensity, 11 studies were based on the traditional cut-point method of acceleration signal thresholding, 5 were metabolic equivalents (MET)-based classifications, and 2 used ENMO (mg)-based classifications (5). In terms of outcome metrics, 18 papers covered multiple types of variables closely related to cardiovascular health, including clinical events (e.g., CVD incidence, myocardial infarction, ischemic stroke), metabolic indicators (e.g., glucose, insulin, HbA1c, lipids, etc.), and markers of inflammation and vascular function (e.g., CRP, IL−6, GDF−15, intra-arterial diameter, etc.), which forms the empirical basis for systematic analyses of the impact of isochronous behavioral substitution effects on cardiovascular health. Table 2 summarizes the basic characteristics of the included literature.

**Table 2:** Study characteristics of included articles.

Author (Year)	Country	Study design	Sample size (n)	Mean age	Measurement method	PA classification	ISM model type	Cardiovascular outcomes measured	Time-use measure	Substitution contrast(s)	Key ISM findings (direction; effect size)
Ryan et al. (36)	UK	Cross-sectional	93	73.8	GENEActiv accelerometer (thigh-mounted, 7 days)	SB: seated/reclined ≤1.5 METs; LIPA: upright 1.5–3.0 METs; sMVPA: ≥3.0 METs <10 min; 10MVPA: ≥3.0 METs ≥10 min	Standard ISM (linear regression, covariate adjusted)	Total cholesterol, Triglycerides, Glucose, HbA1c, IL-6, LPL, PIIINP	Mixed time groups	Multiple reallocations (SB/LIPA/MVPA; varied bouts)	Multiple outcomes/time reallocations were reported
Ryan et al. (16)	UK	Cross-sectional	93	73.6	GENEActiv accelerometer (thigh-mounted, 7 days)	SB ≤1.5 METs, LIPA: 1.5–3 METs, sMVPA: 3–6 METs (1–10 min), 10MVPA ≥3 METs (≥10 min bouts)	Compositional ISM (CoDA, ILR transform)	Triglycerides, Total cholesterol, Glucose, HbA1c, LPL, IL-6, PIIINP	60 min/day	SB -> MVPA (CoDA/ISM)	60 min/day: SB->MVPA associated with lower triglycerides (∼-1.89 to −2.03 mmol/L).
German et al. (41)	USA	Cross-sectional	1,718	68	Actiwatch Spectrum (wrist-worn, 7-day, 24-h wear)	SB: <178.5 cpm; LIPA: 178.6–562.4 cpm; MVPA: >562.4 cpm; sleep scored by algorithm and diary-assisted editing	Standard ISM (linear regression, total time constraint, 30-min substitution)	CVH, SBP, DBP, BMI, Total cholesterol, HDL, LDL, Glucose	30 min/day	SB -> MVPA (standard ISM)	30 min/day: SB->MVPA associated with lower SBP (−1.205 mmHg) and DBP (−0.858 mmHg).
Mellow et al. (10)	Australia	Cross-sectional	397	65.5	Self-reported via Questionnaire (2 × 24-h recalls)	Intensity-based: Sleep (≤0.9 METs), SB (>0.9–1.5 METs), LPA (1.5–2.9 METs), MVPA (>2.9 METs)	Compositional ISM (CoDA, ILR transform)	Waist-to-hip ratio, Systolic BP, Diastolic BP, Total cholesterol	Mixed time groups	Sleep/SB/LPA/MVPA reallocations (CoDA; self-report)	Multiple outcomes/time reallocations were reported
Ryan et al. (35)	UK	Cross-sectional	93	73.8	GENEActiv triaxial accelerometer (thigh-mounted, 7 days)	SB: Seated/reclined posture ≤1.5 METs; LIPA: 1.5–3.0 METs; sMVPA: ≥3.0 METs, <10 min; 10MVPA: ≥3.0 METs, ≥10 min	Standard ISM (forced-entry linear regression, total PB controlled)	Resting heart rate, Carotid artery diameter, Popliteal artery diameter	60 min/day	SB -> standing/MVPA/LPA (standard ISM)	60 min/day: SB->standing RHR −6.20 bpm; SB->MVPA RHR −3.72 bpm; SB->LPA carotid diameter −0.54 mm (95% CI −1.00 to −0.07).
Dumuid et al. (14)	Australia	Cross-sectional	122	65.4	ActiGraph GT3X + accelerometer (waist-worn, 24 h, 7-day)	Freedson cut-points: SB: 0–99 cpm; LPA: 100–1,951; MPA: 1,952–5,274; VPA: ≥5,275; sleep by diary + trace	Compositional ISM (CoDA, ILR transform)	VO2max, BMI, Waist-to-hip ratio, Systolic BP, Diastolic BP, Total cholesterol, Fasting glucose	Mixed time groups	SB -> MVPA (CoDA/ISM; 15-min block)	15 min: SB->MVPA VO2max +1.1 (95% CI 0.2 to 1.9); BMI −0.7 (95% CI −1.0 to −0.3); WHR −1.2 (95% CI −1.8 to −0.7).
Henson et al. (34)	UK	Cross-sectional	372	66.8	ActivPAL3 (thigh-worn, 24 h, 7-day continuous monitoring)	Posture-based classification: Sitting, Standing, Light Stepping (<3 METs), MVPA Stepping (≥3 METs)	Standard ISM (forced-entry linear regression, 60 min substitution)	IL-6, CRP, Leptin (all markers of low-grade inflammation)	60 min/day	Sitting (SB) -> standing/MVPA stepping (standard ISM)	60 min: SB->standing IL-6 −4% (95% CI −7% to −1%); SB->MVPA stepping IL-6 −16% (95% CI −28% to −10%), CRP −41% (95% CI −75% to −8%), leptin −24% (95% CI −34% to −12%).
Yates et al. (37)	UK	Prospective cohort	647	65.0	ActiGraph GT3X accelerometer (waist-worn, 7-day)	SB: <100 cpm; LPA: 100–1,951 cpm; MVPA: ≥1,952 cpm (Freedson cut-points)	Standard ISM (12-month change models, GEE approach)	Waist circumference, 2-h glucose, Triglycerides, HDL, HbA1c, SBP, CMRS	30 min/day	SB -> MVPA (standard ISM; cohort)	30 min/day: SB->MVPA associated with waist circumference −1.23 cm (95% CI −1.79 to −0.68) and CMRS −0.07 (95% CI −0.11 to −0.04).
Nilsson et al. (45)	Sweden	Cross-sectional	113 (female only)	67.5	ActiGraph GT3X (hip-worn, 60 s epoch, ≥4 days, 10 h/day)	SB: <100 cpm; LPA: 100–2,019 cpm; MVPA: ≥2020 cpm; Also calculated: sedentary bouts ≥10 min, break rate	Standard ISM (linear regression with 10-min blocks)	Clustered metabolic risk score (zMS, zMS-wc), Waist circumference, SBP, DBP, Triglycerides, HDL-C, Glucose	10 min/day	MVPA -> LPA/SB (10-min blocks)	10 min: replacing MVPA with LPA/SB associated with higher metabolic risk (zMS +0.06, 95% CI 0.01 to 0.10) and larger waist circumference (+2.19 cm, 95% CI 1.45 to 2.93).
Nilsson et al. (44)	Sweden	Cross-sectional	111 (female only)	67.5	ActiGraph GT3X (hip-worn, ≥4 days, 10 h/day wear time)	SB: <100 cpm; LPA: 100–2,019 cpm; MVPA: ≥2,020 cpm (Freedson)	Standard ISM (linear regression, 30-min substitution)	CRP, Fibrinogen, Adiponectin (systemic inflammation markers)	30 min/day	SB -> MVPA (30-min replacement)	30 min/day: SB->MVPA associated with lower systemic inflammation markers (CRP, fibrinogen); effect sizes not extracted here.
Yates et al. (38)	UK	Cross-sectional	508	65.0	ActiGraph GT3X (hip-worn, 7-day, 15 s epoch, ≥4 valid days)	SB: <25 c/15 s; LPA: 25–487 c/15 s; MVPA: ≥488 c/15 s (Freedson); sensitivity cut at ≥26° c/15 s	Standard ISM (linear regression, adjusted)	HOMA-IS, Matsuda-ISI, Fasting glucose, 2-h glucose, Fasting insulin, 2-h insulin	30 min/day	SB -> MVPA (standard ISM)	30 min/day: SB->MVPA associated with fasting insulin −8% (95% CI −14% to −1%), HOMA-IS +8% (95% CI +1% to +16%), Matsuda-ISI +14% (95% CI +4% to +25%).
Full et al. (40)	USA	Cross-sectional	3,329 (female only)	78.9	ActiGraph GT3X + (hip-worn, 24 h/day, 7-day), sleep log-assisted	SB: <19 c/15 s; LIPA: 19–518 c/15 s; MVPA: ≥519 c/15 s; sleep via Cole-Kripke algorithm	Standard ISM (linear regression, stratified by sleep duration)	Insulin, HOMA-IR, Glucose, Triglycerides, Total cholesterol, HDL, CRP, SBP, Waist circumference, BMI	Mixed time groups	Sleep < -> SB/LPA reallocations (standard ISM)	Mixed: long sleepers reallocate 74 min sleep->LPA: insulin −9.6% (95% CI −14.7% to −4.2%), CRP −15.0% (95% CI −21.5% to −7.8%), TG −6.6% (95% CI −9.6% to −3.5%); short sleepers 91 min SB->sleep: waist −1.3% (95% CI −2.0% to −0.6%), BMI −1.8% (95% CI −2.7% to −1.0%).
Hamer et al. (33)	UK	Cross-sectional	445	66.0	ActiGraph GT3X (hip-worn, 4–7 days, waking hours only)	SB: 0–199 cpm; LPA: 200–1,998 cpm; MVPA: ≥1,999 cpm (based on METs: <1.5, 1.5–3, ≥3)	Standard ISM (linear regression, 10-min substitution)	HbA1c, BMI, HDL-C, Triglycerides (cardiometabolic risk profile)	10 min/day	SB -> MVPA (10-min blocks)	10 min/day: SB->MVPA HbA1c −0.023 (95% CI −0.043 to −0.002), BMI −0.39 (95% CI −0.54 to −0.24), TG −0.035 (95% CI −0.061 to −0.009), HDL +0.037 (95% CI 0.021 to 0.054).
McGregor et al. (46)	Canada	Cross-sectional	1,454	(65–79 age group	Actical accelerometer (hip-worn, 7-day, 60 s epoch); sleep via self-report	SB: <100 cpm; LPA: 100–1,534 cpm; MVPA: ≥1,535 cpm; Sleep self-reported in hours	Compositional ISM (CoDA, ILR transform)	BMI, Waist circumference, Aerobic fitness, SBP, DBP, Resting heart rate, HDL, LDL, Triglycerides, Glucose, Insulin, CRP	Mixed time groups	SB/LPA/MVPA/sleep reallocations (CoDA; sleep self-report)	Multiple outcomes/time reallocations were reported
Ortolá et al. (47)	Spain	Cross-sectional	2,245	71.6	ActiGraph GT9X (wrist-worn, 7-day, 24 h), sleep by algorithm	SB: <45 mg; LPA: 45–99 mg; MVPA: ≥100 mg (ENMO cut-points)	Standard ISM (linear regression, 30-min replacement)	GDF-15 (growth differentiation factor 15–cardiovascular-related inflammatory biomarker)	30 min/day	SB -> MVPA (30-min replacement)	30 min/day: SB->MVPA associated with lower GDF-15 (inactive −14.7%, 95% CI −18.6% to −10.7%; active −3.5%, 95% CI −5.8% to −1.1%).
Yerramalla et al. (39)	UK	Prospective cohort	3,319	68.9	GENEActiv wrist-worn accelerometer (24 h, 7-day), GGIR package	SB: <40 mg; LIPA: 40–99 mg; MVPA: ≥100 mg (based on ENMO, mg)	Compositional ISM (Cox regression, ILR transform)	Incident CVD (CHD, stroke, heart failure via ICD codes and registry)	Mixed time groups	SB/LIPA/MVPA reallocations (CoDA; Cox)	Multiple outcomes/time reallocations were reported
Peter-Marske et al. (43)	USA	Prospective cohort	16,031 (female only)	71.4	ActiGraph GT3X + (hip-worn, 7-day, 10 h/day valid wear)	SB: <19 VM/15 s; HLPA: 226–518 VM; MVPA: ≥519 VM (age-calibrated thresholds)	Standard ISM (Cox regression, 10–30 min substitution)	Total CVD, Myocardial Infarction (MI), Ischemic Stroke (IS) (adjudicated via records)	10 min/day	SB -> MVPA (10–30 min; Cox)	SB->MVPA associated with lower CVD risk (HR 0.96, 95% CI 0.93 to 0.99; ∼4% lower risk per 10 min).
McAlister et al. (42)	USA	Cross-sectional	54	72.7	ActiGraph GT3X (hip-worn, 7-day, ≥8 h/day on ≥4 days)	SB: <100 cpm; LPA: 100–2,019 cpm; MVPA: ≥2,020 cpm (Troiano cut-points)	Standard ISM (linear regression, 10-min substitution)	BMI, Waist circumference, SBP, DBP, HDL, Total cholesterol, Non-HDL cholesterol, Fasting glucose	10 min/day	SB -> LPA (10-min blocks)	10 min/day: SB->LPA DBP −0.472 mmHg (95% CI −0.85 to −0.09); HDL +0.843 mg/dL (95% CI −0.01 to 1.70).

SB, sedentary behavior; LPA, light physical activity; MVPA, moderate-to-vigorous physical activity; sMVPA, sporadic moderate-to-vigorous physical activity; 10MVPA, MVPA in ≥10-min Bouts; MET, metabolic equivalent of task; cpm, counts per minute; ENMO, euclidean norm minus one; VM, vector magnitude; ISM, isotemporal substitution model; CoDA, compositional data analysis; ILR, isometric log-ratio; HbA1c, glycated hemoglobin; HDL, high-density lipoprotein; LDL, low-density lipoprotein; CRP, C-reactive protein; IL-6, interleukin-6; GDF-15, growth differentiation factor 15; HOMA-IR, homeostatic model assessment of insulin resistance; HOMA-IS, homeostatic model assessment of insulin sensitivity; VO_2max_, maximal oxygen consumption; CVD, cardiovascular disease; CHD, coronary heart disease.

.

### 3.3 Risk of bias assessment

Of the 18 papers included in this study, 15 were analytic cross-sectional studies and 3 were prospective cohort studies. Overall, methodological quality was assessed using the Joanna Briggs Institute (JBI) critical appraisal checklists, and item-level judgments for each study are reported in Supplementary Tables S1 (cohort) and S2 (cross-sectional).

On the one hand, for cross-sectional studies, we considered studies meeting at least 6 of the 8 JBI items as methodologically acceptable, and studies meeting 7 or more items as high quality. All 15 cross-sectional studies met at least 6 items, with 13 meeting 7 or more items, suggesting an overall low risk of bias. On the other hand, for cohort studies, we considered studies meeting 9 or more of the 11 JBI items as high quality. All three cohort studies fulfilled 9 of 11 items and were therefore judged to be of high methodological quality.

Across study designs, the most common limitations were incomplete reporting or handling of confounding (cross-sectional checklist items on identifying confounders and specifying strategies) and incomplete follow-up–related items in cohort studies. Nevertheless, given that all included studies met the predefined quality thresholds, the overall risk of bias was considered low, supporting the reliability of the narrative synthesis.

### 3.4 Effects of isotemporal substitution on cardiovascular outcomes

In order to better understand the effects of substituting PA for SB on cardiovascular health in older adults, this section summarized and integrated the main findings of the included studies along the following two dimensions. First, to explore the effects associated with substituting sedentary time (Sedentary Behavior, SB) for physical activity (Physical Activity, PA) on the cardiovascular health of older adults; and second, to collate the effects of substituting different levels of physical intensity (light PA vs. moderate to high intensity PA) on the cardiovascular health of older adults.

#### 3.4.1 Effects of time reallocation on cardiovascular health

The role of different isochronous alternative times on cardiovascular health in older adults shows significant heterogeneity in existing studies. The 18 studies included in this paper cover alternative pathways at different times (see Table 2. The studies generally used accelerometers to measure behavioral time and used the isochronous substitution model (ISM) to estimate the effect of time reallocation between multiple activity types on cardiovascular indices. Depending on the duration of substitution, the findings can be summarized as follows:

a) 10 min/day. Replacing daily 10-min SB with MVPA was associated with more favorable cardiovascular metabolic indices in older adults, including significant reductions in HbA1c (–0.023, 95% CI: −0.043 to −0.002), BMI (–0.39, 95% CI: −0.54 to −0.24), and triglyceride levels (–0.035, 95% CI: −0.061 to −0.009) and elevated HDL-C (+0.037, 95% CI: 0.021–0.054) (33); furthermore, a 10-min SB replacement with MVPA was related to a lower CVD risk in older adults by approximately 4% (HR: 0.96, 95% CI: 0.93–0.99) (43), whereas LPA replacement for SB mainly was observed with improved diastolic blood pressure (–0.472 mmHg, 95% CI: −0.85 to −0.09) and HDL-C (+0.843 mg/dL, 95% CI: −0.01–1.70) (42); notably, metabolic risk metrics worsened significantly when MVPA was replaced by LPA or SB, such as aggregated metabolic risk score increase (zMS: +0.06, 95% CI: 0.01–0.10) and waist circumference increase (+2.19 cm, 95% CI: 1.45–2.93) (45). These studies suggest that even relatively short activity substitutions can accompanied by clinically meaningful cardiovascular protective associations, but that there is a clear intensity threshold for such effects and that the loss of MVPA's protective associations may be related to rapid metabolic deterioration, which has important implications for the design of health promotion strategies for older adults.
b) 30 min/day. Increasing the duration of substitution to 30 min/day began to show broader cardiovascular benefits than the 10-min substitution pathway, e.g., substitution of 30-min SB for MVPA was observed with lower systolic blood pressure (–1.205 mm Hg) and diastolic blood pressure (–0.858 mm Hg) (41), and was accompanied by reduced C-reactive protein (–0.35, 95% CI: −1.11 to −0.02) and fibrinogen (–0.31) in older women (41), as well as was linked to lowering C-reactive protein (–0.35, 95% CI: −1.11 to −0.02) and fibrinogen (–0.31, 95% CI: −1.11 to −0.01) in older women (44); In older adults at higher metabolic risk, 30-min SB replacement with MVPA showed associations with improved insulin sensitivity metrics, including reduced fasting insulin (–8%, 95% CI: −14% to −1%), increased HOMA-IS (+8%, 95% CI: +1% to +16%) and Matsuda-ISI (+14%, 95% CI: +4% to +25%) (38), and these improvements in insulin action may have promoted a reduction in waist circumference (–1.23 cm, 95% CI: −1.79 to −0.68) and cardiometabolic risk score (–0.07, 95% CI: −0.11 to −0.04) were reduced (37); of particular note, the cardiovascular aging marker GDF-15 was related to reduced by substitution of SB for MVPA in older adults with low levels of activity (–14.7%, 95% CI: −18.6% to −10.7%), while the effect was attenuated in individuals with high activity levels (–3.5%, 95% CI: −5.8% to −1.1%) (47). Across the included studies, the findings at the 30-min window are consistent with a dose-response pattern in observational estimates, and the 30-min reallocation represents a commonly examined duration at which broader associations across cardiovascular and metabolic indicators become apparent; associations also appeared more pronounced in higher-risk subgroups, which may help contextualize intensity- and risk-stratified recommendations while avoiding a causal or prescriptive interpretation.
c) 60 min/day. The 60-min replacement model was associated with more comprehensive cardiovascular benefits, with significant improvements in several cardiovascular functions, including lipid metabolism, e.g., the replacement of SB with MVPA was linked to lower triglycerides by approximately 1.89–2.03 mmol/L (16). Also, vascular structure and function showed significant adaptations, replacing 60-min SB with standing or MVPA reduced resting heart rate (–6.20 bpm and −3.72 bpm, respectively), and replacing SB with LPA was related to reduced carotid artery diameter (–0.54 mm, 95% CI: −1.00 to −0.07) (35). In addition, the inflammatory response to 60-min substitution showed a clear intensity-dependent pattern, with Standing substitution for SB was accompanied by moderately reducing IL-6 (–4%, 95% CI: −7% to −1%), whereas MVPA stepping was linked to a more comprehensive anti-inflammatory effect, a significantly lowering IL-6 (–16%, 95% CI: −28% to −10%), CRP (–41%, 95% CI: −75% to −8%), and Leptin (–24%, 95% CI: −34% to −12%) (34). Overall, the 60-min reallocation pathway was linked to broader cardiovascular profiles and clearer intensity-related contrasts, suggesting that longer substitutions may be feasible for some older adults; these observations can help shape the rationale for future longitudinal and intervention studies, while not establishing causal effects or an “optimal” duration.
d) Mixed time groups. In addition to standardized time blocks, some studies have used component data analysis or variable replacement duration to provide additional insights into the relationship between behavioral reallocation and cardiovascular health. These approaches suggest that the cardiovascular associations of activity replacement may be moderated by baseline activity levels, with larger estimated differences observed in inactive individuals (36). In terms of the interaction between sleep duration and activity replacement, for long sleepers, reallocation of excess sleep time (74 min) to LPA was associated with statistically significant differences in several cardiometabolic markers, including insulin (–9.6%, 95% CI: −14.7% to −4.2%), CRP (–15.0%, 95% CI: −21.5% to −7.8%), and triglycerides (–6.6%, 95% CI: −9.6% to −3.5%), while for short sleepers, replacing SB with (91 min) sleep improved body composition measures, including reductions in waist circumference (–1.3%, 95% CI: −2.0% to −0.6%) and BMI (–1.8%, 95% CI: −2.7% to −1.0%) (40). In addition, component isochronous substitution modeling further demonstrated that reallocating only 15 min of SB to MVPA significantly improved cardiorespiratory fitness (VO_2max_: +1.1, 95% CI: 0.2–1.9), BMI (–0.7, 95% CI: −1.0 to −0.3), and waist-to-hip ratio (–1.2, 95% CI: −1.8 to −0.7) (14). These innovative methodological studies break through the limitations of traditional isochronous substitution models and reveal the complex interactions between behaviors in the 24-h activity cycle, especially the bidirectional relationship between Sleep and PA, a finding that is an important guide for the optimization of all-weather activity patterns in older adults.

#### 3.4.2 Effects of substitution intensity on cardiovascular health

In empirical studies of isochronous substitution models, behavioral intensity has been identified as a key variable related to cardiovascular health outcomes in older adults. Compared to time reallocation alone, isotemporal substitution of different intensities of physical activity (PA) is associated with differences in physiological load and metabolic regulatory responses per unit of time, which may help explain observed differences in blood pressure and lipids, cardiovascular metabolism, and cardiovascular aging indices.

a) Cardiovascular function. First, in terms of blood pressure regulation, MVPA replacing SB was associated with lower systolic blood pressure (–1.205 mmHg) and diastolic blood pressure (–0.858 mmHg) in the elderly, whereas LPA replacing SB not only had a limited ameliorative effect, but may even have caused an increase in diastolic blood pressure (+0.147 mmHg) in the elderly (41). This pattern suggests that higher-intensity substitutions may show more favorable profiles in relation to BP indicators in older adults. Second, vascular structural adaptations also show specific responses to physical activity intensity, e.g., substituting LPA for SB reduces carotid artery diameter (–0.54 mm), whereas substituting Standing for sMVPA was observed with a larger popliteal artery diameter (+1.31 mm) (35). Additionally, cardiac autoregulation (reflected as resting heart rate) showed stronger estimated differences in higher-intensity substitution, with MVPA being more effective than LPA in reducing resting heart rate in older adults, suggesting that high-intensity physical activity enhances parasympathetic tone and cardiovascular efficiency in older adults (35, 46). This intensity-dependent pattern of blood pressure and vascular responses reveals that high-intensity activity may produce more significant cardioprotective effects through enhanced endothelial function and autonomic modulation, a mechanistic difference that could be valuable in understanding exercise prescription for cardiovascular health in older adults.
b) Lipid metabolism. A series of studies have shown that the effects of different intensity activity substitutions on lipid metabolism in older adults exhibit a clear gradient effect, with the MVPA substitution pathway consistently linked to lower triglyceride levels in multiple studies, with effect sizes ranging from −0.035 mmol/L for the 10-min substitution to between–1.89 and −2.03 mmol/L ranging for the 60-min substitution (16, 33, 37, 46). Meanwhile, HDL cholesterol exhibited a similar intensity-dependent response, with MVPA associated with higher HDL-C levels (+0.037 mmol/L, 95% CI: 0.021–0.054), whereas LPA replacement produced small or non-significant changes (33). However, related studies have also noted that total cholesterol in older adults may be observed to be higher with LPA replacement (+0.534 mg/dL) while MVPA replacement remains unchanged (41). This intensity threshold phenomenon of lipid metabolism suggests that there may be a minimum intensity of activity beyond which only lipid metabolism-related enzyme systems and signaling pathways can be effectively activated, which has important practical implications for nonpharmacological intervention strategies for lipid abnormalities in older adults.
c) Glucose metabolism and insulin sensitivity. Activity intensity showed a significant threshold effect on glucose metabolism and insulin sensitivity in older adults. It was noted that SB replacement with MVPA was associated with more favorable markers of insulin sensitivity, including HOMA-IS (+1.08, 95% CI: 1.01–1.16) and Matsuda-ISI (+1.14, 95% CI: 1.04–1.25), while dramatically decreasing 2-h insulin levels (–15%, 95% CI: −23% to −6%) (38). In particular, when LPA was replaced with MVPA, it further reduced 2-h glucose (–0.16 mmol/L, 95% CI: −0.30 to −0.02) and cardiometabolic risk score (–0.05, 95% CI: −0.09 to −0.01) (37). It is important to note that metabolic markers deteriorated rapidly when MVPA was replaced by LPA or SB, with significant increases in aggregated metabolic risk score (zMS) and waist circumference (WC) (45). This intensity-dependent pattern of metabolic responses reveals that high-intensity activity may overcome the insulin-resistant state common in older adults through more effective activation of the insulin signaling pathway and glucose transporter protein system, which could be of great clinical value for exercise interventions for prediabetes and metabolic syndrome in the elderly.
d) Inflammation and aging biomarkers. The intensity-dependence of the inflammatory response has been consistently confirmed in several studies, and while both LPA and MVPA replacement were associated with lower inflammatory markers, MVPA was generally linked to larger and more comprehensive anti-inflammatory effect (34). Furthermore, the cardiovascular aging biomarker GDF-15 similarly demonstrated an intensity-dependent response, with MVPA replacement associated with larger reductions in individuals with low activity levels (–14.7%, 95% CI: −18.6% to −10.7%) compared to LPA replacement (–6.2%, 95% CI: −8.8% to −3.5%) (47). Of interest, the effect of MVPA replacement on inflammatory markers was particularly significant in a cohort study of older women (44). This intensity-dependent response in markers of inflammation and aging suggests that high-intensity activity may attenuate the chronic low-grade inflammatory state common in older adults through more effective activation of anti-inflammatory pathways and antioxidant systems, which has important implications for delaying cardiovascular aging and reducing the risk of inflammation-related diseases in older adults.
e) Body composition and CVD risk. The response of body composition to activity intensity showed a significant gradient effect, with MVPA replacement generally associated with larger reductions in BMI, waist circumference and waist-to-hip ratio in several studies compared to LPA replacement of equivalent duration, e.g., reallocating 15 min to MVPA was associated with lower BMI(0.7 kg/m^2^) and waist-to-hip ratio (1.2%), whereas the same amount of time spent in LPA replacement produced no significant change in these metrics (14); and 30-min MVPA replacement was observed with a 1.23 cm lower waist circumference, compared to only 0.21 cm with equivalent LPA replacement (37). Furthermore, the effect of activity intensity on cardiovascular clinical outcomes demonstrated a clear threshold effect, with SB replacement with MVPA significantly reducing CVD risk, whereas HLPA replacement did not show a significant risk reduction (39, 43). These studies suggest that high-intensity activity may be enhanced by greater energy expenditure and more significant post-exercise metabolic rate, and that activity intensity may be a key factor not to be overlooked in order to achieve significant cardiovascular risk reductions, which could be an important guideline for the development of CVD prevention strategies in older adults.

## 4 Discussion

### 4.1 Summary of main findings

**Figure 2:**
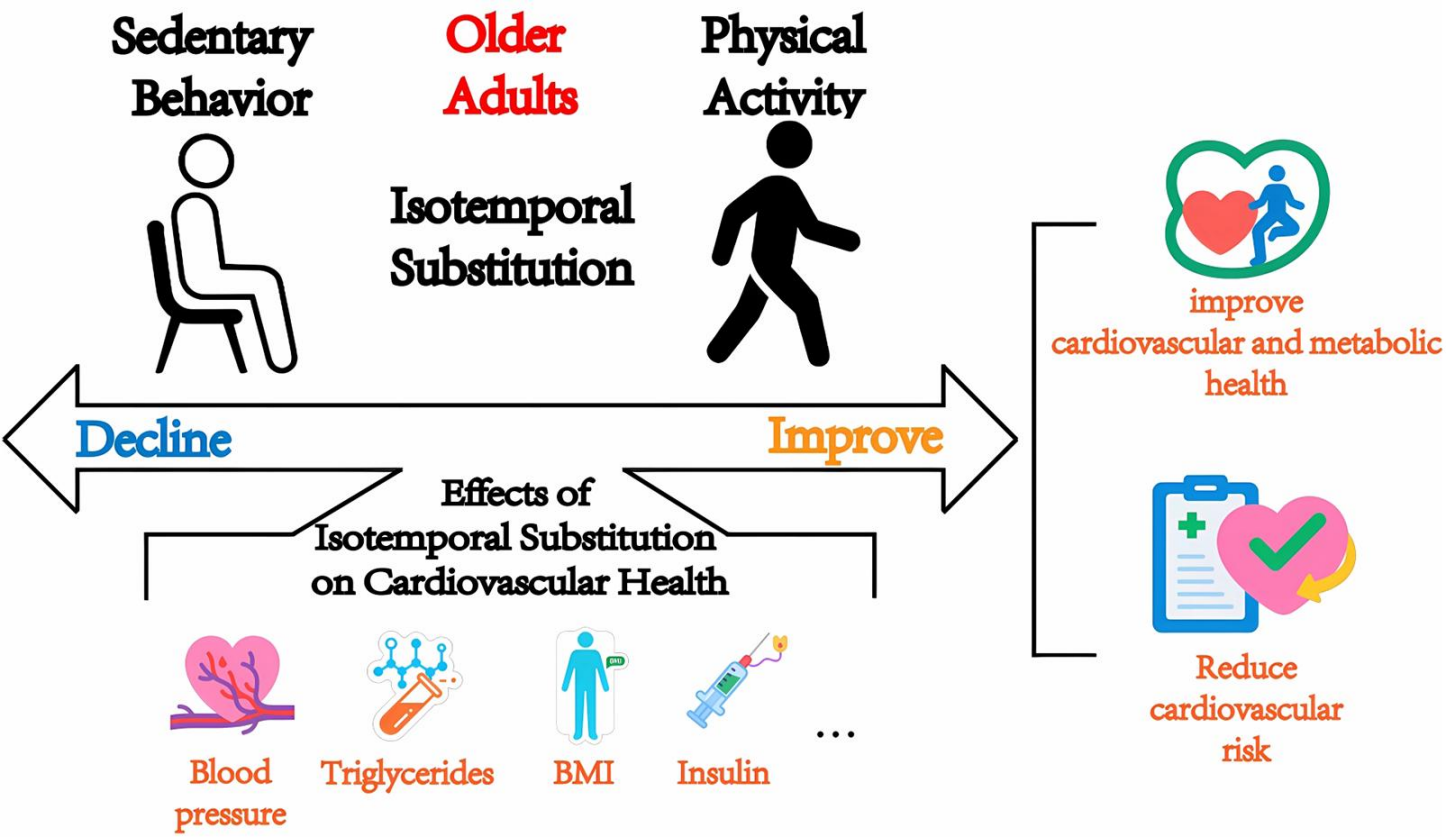
Mechanism of replacing sedentary behavior with physical activity on cardiovascular health in the elderly.

This systematic review provides a comprehensive assessment of the associations between sedentary behavior and physical activity time reallocation and cardiovascular health in older adults. Based on a comprehensive synthesis of 18 observational studies, with generally low risk of bias per JBI appraisal, we found that the isochronous substitution model (ISM) provides a robust methodological framework for understanding the health-related patterns of behavioral time reallocation, providing a structured way to examine the reported associations between time reallocation (e.g., replacing SB with PA) and cardiovascular health indicators in an older population (Figure 2). For example, in accelerometer-based ISM analyses, reallocating 10 min/day of SB to MVPA was estimated to be associated with lower HbA1c (−0.023, 95% CI: −0.043 to −0.002) (33) and a modestly lower CVD risk estimate (HR: 0.96, 95% CI: 0.93–0.99) (43). These values should be interpreted as observational time-reallocation estimates rather than definitive causal effects, particularly given the predominance of cross-sectional designs and the risk of reverse causation. Notably, most included studies were cross-sectional (15/18), and reverse causation is plausible in older adults, as poorer cardiometabolic health, higher inflammatory burden, frailty, or functional limitations may lead to less MVPA and more sedentary time, potentially strengthening observed SB-PA associations. Accordingly, the findings in this review should be viewed as evidence of reported associational patterns that appear relatively consistent for some outcomes, while causal inference requires longitudinal designs with repeated measures and stronger control of confounding.

To respond in depth to the first research question (RQ1), focus was placed on differential trends in the cardiovascular health associations of replacing SB with PA in older adults at different isochronous substitution lengths. First, during short SB replacement for PA pathways (e.g., 10 or 15 min), research has mostly focused on capturing whether low-dose behavioral switching can be associated with detectable changes in bodily systems. While some studies have reported significant improvements in DBP or Total CDV with physical activity substitution for SB (14, 42), there are also studies that have not observed significant changes or have shown marginal trends (39, 43). The importance of this type of alternative pathway may not lie in its immediate effectiveness, but rather in providing a minimal behavioral alternative unit that is more consistent with the reality of older adults and has a high degree of life feasibility. Second, the 30-min alternative, the most dominant modeling setting, has been shown in several studies to illustrate more consistently favorable associations on cardiovascular health indicators in older adults and may constitute a practical reference window for behavior reallocation (37, 41, 47). Furthermore, in alternative pathways that were higher than 60 min or set to multiple time points, the findings showed more variability. While it is true that expanding the duration of substitution may result in more metric improvements (16), this effect does not occur consistently across all studies (35, 40), and some combinatorial modeling pathways have also pointed to the fact that temporal distributions of physical activity substitutions for sedentary behaviors collectively may be related to older adults’ cardiovascular health wellness marginal effects (46). Thus, different temporal substitution pathways are not simply incremental in their effects on cardiovascular health in older adults, but more like a search for feasible time nodes from multiple response compartments. While most studies consider 30 min as constituting a critical unit for stable response and 60 min as an extended unit for behavioral shaping, it is important to emphasize that the value of different time-alternative effects on cardiovascular health in older adults also shows dynamic differences depending on older adults’ physical status, activity intensity, and activity behavior.

Centered around the second research question (RQ2), the numerous studies included in this study demonstrated significant differences in the intervention effects of different intensities of physical activity on cardiovascular health in older adults when replacing sedentary behaviors. Compared to light physical activity (LPA), moderate to vigorous intensity activity (MVPA) has more consistently demonstrated significant favorable associations with core cardiovascular metrics such as insulin sensitivity, waist circumference, triglycerides, and inflammatory markers (38, 41, 46). This difference may not stem solely from a physiological role gap in the magnitude of physical activity intensity, but rather reflects the behavioral attributes and structural functions attached to physical activity intensity. For example, research suggests that MVPA is more likely to constitute planned, active exercise, which typically occurs with higher metabolic load, behavioral motivation, and self-regulation, and thus is more advantageous in terms of behavioral consistency and benefit accumulation, whereas LPA tends to manifest as background activities in older populations, such as household chores, standing, or slow walking, with fragmentation characteristics that limit its sustained association with stable cardiometabolic profiles (48). More importantly, the MVPA pathway is more likely to form a series of behavioral chain structures, i.e., its substitution effect is often not only a hedge against SB, but also accompanied by indirect gains such as sleep structure optimization and daytime function modulation, thus acting synergistically in the behavioral system as a whole (49). Moreover, on a methodological level, MVPA studies are also more goal-oriented in their design, whereas LPA pathways tend to be limited by vaguely defined activity classification criteria, measurement instruments, and behavioral contexts, which partly explains the lack of consistency in their results (39). However, it is also important to emphasize the greater behavioral accessibility and practicality of LPA for older or slow patient populations who have difficulty with MVPA activities, creating a cumulative cumulative effect of sustained engagement in LPA on alternative SBs, which can lead to trend improvements or significant effects on cardiovascular health in older adults (50). Therefore, the alternative pathway of MVPA to SB has better relevance to the cardiovascular health of older adults, not only because of its higher energy consumption, but also because of its behavioral level of goal-focusedness, stability of implementation, and systemic linkage, while the complementary role of LPA cannot be ignored.

In conclusion, this study systematically investigated the cardiovascular health effects of different isochronous substitution time lengths and different intensities of physical activity in replacing sedentary behaviors in older adults, and revealed that the MVPA pathway has better health benefits due to its high metabolic load and stable behavioral structure, and at the same time, emphasized that the LPA, as a more accessible form of intervention, should not be ignored, which provides a structured synthesis to inform future research and cautiously framed practice considerations for older adults. By consolidating ISM-based observational evidence specifically in adults aged ≥65 years and organizing the findings by substitution duration and intensity (LPA vs. MVPA), this review offers a practical lens for interpreting a literature that is otherwise dispersed across designs, metrics, and modeling choices. In particular, it helps distinguish patterns that appear comparatively more consistent from those that remain context-dependent—most notably for LPA—thereby providing a clearer empirical basis for shaping future longitudinal study priorities and for supporting cautious, age-adapted guideline discussions in ageing societies. It also lays a scientific foundation for the development of more targeted and operable cardiovascular health promotion programs, while acknowledging that causal claims require stronger longitudinal evidence.

### 4.2 Comparison with existing reviews

This study systematically reviewed the effects of isochronous substitution of SB to PA of different intensities on cardiovascular health in the elderly population, deepened the theory and expanded the empirical evidence of the existing literature in terms of the focus of the study population, the construction of substitution pathways, and the integration of indicators of physiological mechanisms, and formed a clear contrasting difference with the current mainstream review studies.

First, compared with previous studies that focused on adults or mixed-age groups (51, 52), the present study explicitly focuses on older adults aged 65 years and older, filling the gap in evidence on the association between sedentary replacement behaviors and cardiovascular health in this population, and reinforcing the relevance of the study for policy translation. Meta-analyses in middle-aged adults have shown that MVPA replacement of SB is effective in improving metabolic syndrome indicators (53), but most of these studies have ignored the context of physiological deterioration and limitations in exercise function that are unique to older adults. In addition, a large accelerometer-based prospective cohort reported inverse associations between MVPA and incident cardiometabolic outcomes, providing complementary longitudinal context beyond the time-reallocation studies synthesized here (54). In contrast, the observational studies included in this study clearly demonstrate the positive effects of SB replacement by MVPA or LPA on cardiovascular health in older adults, which further enriches and complements the existing multidimensional and significant effects of SB replacement by PA in older adults in terms of physical functioning, mental health, and frailty prevention (55–57), providing empirical evidence based on a behavioral substitution perspective for the future development of cardiovascular health promotion strategies for older adults.

Second, in terms of research methodology, this study systematically adopted the Isotemporal Substitution Model (ISM), a modeling approach that has seen limited use in the existing review literature. Although Panahi and Tremblay (58) and Bull et al. (21) mentioned the relationship between SB and PA in their respective studies, they mostly failed to carry out research on the relationship between SB substitution of PA in “time-dose” and “behavior-intensity” comparisons. In the present study, we further integrated the dose-response relationships of 10, 30, and 60 min of substitution, and found that MVPA replacing 30 min of SB was associated with the most significant gains in several cardiovascular physiological indices (e.g., SBP, DBP, and triglycerides), which echoes Richardson et al.'s (59) findings on the relationship between MVPA and markers of inflammation (e.g., fibrinogen and PAI-induced inflammation), thus enhancing the explanatory power of isochronous alternative mechanisms).

Again, unlike previous reviews that have less often touched on or marginalized the health value of LPA (60), the present study systematically reviewed the protective effects of LPA replacement with SB in an elderly, physically limited group. In a meta-analysis, an additional hour of LPA per day was associated with a 10% reduction in the risk of cardiovascular events in older adults (61), whereas Kim et al. (50) found that replacing SB with LPA alone, even in the absence of MVPA, reduced all-cause and cardiovascular mortality by 26% and 24%, respectively. The present study integrates the above marginal evidence into a behavioral substitution system, suggests the value of “split-intensity substitution strategies” for older adults with different mobility, and expands the accessibility and adaptability of cardiovascular exercise interventions for older adults.

### 4.3 Strengths and limitations

The present study provides a systematic and comprehensive review of evidence for understanding the associations of isochronous substitution between SB and PA with cardiovascular health in older adults, with significant theoretical and practical contributions. First, based on a systematic search and rigorous screening, this study integrated and compared 18 original ISM-based studies, providing a clearly structured and focused summary of the evidence in the field, which can help to identify the consistency and variability of the current studies, and lay the methodological foundation for subsequent research and practice applications. Second, this study analyzed the “alternative time length” and “alternative intensity path” dimensions: on the one hand, the review systematically presented the distribution of health effects under different alternative lengths ranging from 10 min to 60 min and above, and pointed out that 30 min might constitute the most common window for behavioral response. On the other hand, the study clearly compares the differences in cardiovascular metabolism, inflammation, and body composition between the LPA and MVPA pathways, highlighting the more consistent associations for MVPA substitution in terms of magnitude and consistency, and also pointing out the potential for LPA to be realistically adapted to the elderly population in the older age group or in the mobility-constrained population. This two-dimensional path resolution not only clarifies the logical hierarchy of current research on isochronous substitution behavior, but also provides theoretical support and empirical evidence for the development of future research and cautiously framed practice considerations for cardiovascular health in older adults.

Despite the strengths of this study in terms of systematic integration and theoretical construction, there are still several limitations that need to be pointed out. First, most of the included studies were cross-sectional or short-term observational studies, and long-term follow-up data were scarce, making it difficult to comprehensively portray the persistent and cumulative effects of behavioral alternative pathways on cardiovascular health. Accordingly, the ISM-derived estimates should be interpreted as associations rather than definitive causal effects. Importantly, the predominance of cross-sectional designs raises a high risk of reverse causation bias in older adults, because poorer cardiometabolic health, higher inflammatory burden, frailty, or functional limitations may reduce MVPA and increase sedentary time, thereby strengthening observed SB–PA associations; residual confounding (e.g., comorbidity severity, medication use, and functional capacity) may also persist even in adjusted models, further limiting causal interpretation. Second, cross-study methodological heterogeneity may have limited the direct comparability of effect estimates, and thus the synthesis should not be interpreted as implying identical statistical effects across data types, which is particularly relevant for LPA-related findings. Studies varied in how LPA and MVPA were operationalized (e.g., processing decisions and intensity cut-points) and in the behavioral components modeled alongside SB and PA (e.g., whether standing or sleep was explicitly included), which can shift the SB-LPA boundary and may partly explain the inconsistency of LPA substitutions across outcomes and populations compared with MVPA.Third, because we limited inclusion to English-language publications, relevant evidence published in other languages may have been missed, which could introduce language bias and affect the completeness of the evidence base. In addition, we restricted inclusion to peer-reviewed journal articles and applied these limits at the database search stage via platform filters where available, which may have excluded potentially relevant grey literature (such as dissertations) and may contribute to the risk of publication bias, meaning our conclusions primarily reflect the English-language, peer-reviewed evidence base rather than the full spectrum of available research.Therefore, future research would benefit from harmonized measurement and analytic reporting, for example, clearer intensity-definition transparency and more comparable ISM specifications, alongside larger and longer-term cohorts with repeated measures, to improve cross-study comparability—especially for LPA—and to strengthen causal interpretation.

### 4.4 Implications for research and policy

The findings of this systematic review are of great practical value for informing hypothesis-generating practice considerations and future research priorities for older adults. Given that most included studies were cross-sectional, the following implications should be interpreted cautiously as they are based on observational associations that may be subject to reverse causation and residual confounding, rather than definitive causal evidence.

First, based on the regular patterns revealed by isochronous substitution analyses, it is proposed to construct the “10–30–60 min substitution model” as a pragmatic set of reference windows for time reallocation. In this model, a 10-min daily replacement of MVPA can be regarded as a feasible minimal increment frequently examined in the literature, which was often associated with more favorable profiles in insulin sensitivity and inflammatory factors; a 30-min replacement is commonly associated with favorable differences in blood pressure, triglycerides, and other clinical indicators, and can be used as a candidate duration for guideline-relevant evaluation in longitudinal or interventional studies; and a 60-min replacement or more was sometimes linked to broader favorable cardiovascular and metabolic profiles in some studies, although mechanisms and causality cannot be inferred from the current evidence base. The model is highly flexible and scalable, and can be adapted to older populations with different levels of physical ability as a tiered, feasibility-oriented framework that warrants confirmation in stronger causal designs.

Second, the findings of this study highlight potential considerations for future guideline-relevant research in terms of adaptation to the older population. Although the guidelines released by the World Health Organization (WHO) in 2020 recommend 150–300 min of moderate-intensity or 75–150 min of vigorous-intensity physical activity per week for people aged 65 years and older, the risk thresholds for sedentary behavior and the potential compensatory value of light-intensity physical activity (LPA) have not yet been provided with clear Guidance. However, the present study suggests that although the health effects of LPA are weaker compared to MVPA, LPA replacement is potentially relevant from a feasibility perspective in very old, multimorbid, or exercise-limited older adults. Therefore, there is a need for future guidelines to be informed by responsive recommendations for behavioral heterogeneity and functional diversity in older adults, ideally supported by longitudinal evidence that can better address reverse causation.

Third, a shift from static behavioral recommendations to dynamic, individualized exercise strategies should be promoted. This not only requires the integration of sedentary behavior, physical activity, and sleep into the “24-h behavioral profile” for combined analysis, but also relies on wearable technology to achieve real-time tracking of behavioral status and timely feedback, thus improving the accessibility and compliance of alternative behavioral programs. From a research perspective, such monitoring also enables repeated-measure designs that can strengthen causal inference beyond cross-sectional snapshots.

Lastly, the effective implementation of isochronous substitution strategies requires multidimensional support at the policy level, including promoting the design of age-appropriate public environments (e.g., safe walking spaces), enhancing the ability of older adults to use digital technology, and encouraging the implementation of preventive exercise programs through the health insurance mechanism. Only through the organic integration of evidence, policy, and service systems can we truly promote “sedentary substitution behavior” in the direction of precise aging health promotion efforts that are operable, sustainable, and universally beneficial in reality while continuing to update recommendations as stronger longitudinal and experimental evidence becomes available.

## 5 Conclusions

This systematic review synthesised evidence from 18 observational studies (15 cross-sectional and 3 prospective cohorts), most of which were rated as low risk of bias using the JBI critical appraisal checklists, and found that isotemporal replacement of SB with PA, particularly 30–60 min/day of moderate to vigorous physical activity (MVPA), in the elderly population, was associated with lower systolic and diastolic blood pressure, with more favorable lipid profiles (HDL-C, TG), enhanced insulin sensitivity, suppressed low-grade inflammation, and modulated cardiovascular aging biomarker GDF-15; whereas light-intensity activity (LPA), although with relatively limited associations, may offer a feasible pathway to health gains in individuals with physical limitations. To avoid redundancy, the above sentence summarizes the main intensity-specific patterns reported across studies.Meanwhile, dose-response analyses suggest that a minimal substitution of 10 min/day can be associated with measurable differences, while 30 min is the most commonly examined reference window at the clinical and public health level, and 60 min and beyond may trigger synergistic improvements in vascular structure and autonomic regulation. Limitations such as cross-study methodological heterogeneity, inconsistent accelerometer thresholds, and cross-sectional designs limit the extrapolation of causal inferences and refined modeling of behavioral dosage. In particular, given that most evidence was cross-sectional, the observed ISM estimates should be interpreted as associations that may be subject to reverse causation (e.g., poorer cardiometabolic health or functional limitations leading to less MVPA and more sedentary time) and residual confounding, rather than definitive causal effects.Future studies should integrate 24-h behavioral profiles including sleep, long-term multicenter cohorts, and contextualized programs, with a special focus on behavioral accessibility and sustained adherence in elderly and multimorbid populations. In conclusion, this review emphasizes the critical value of “moving more and sitting less” as a public health message relevant to cardiovascular health in older adults, and provides an evidence base to inform guideline-relevant research and the development of feasibility-oriented, tiered recommendations, pending confirmation in stronger longitudinal and experimental designs.

## Data Availability

The original contributions presented in the study are included in the article/Supplementary Material, further inquiries can be directed to the corresponding author.
