# Supplementary material for: Isotemporal substitution of sedentary time with physical activity for cardiovascular health in older adults: a systematic review
Source: Front Sports Act Living. 2026 Feb 25;8:1708003. doi: 10.3389/fspor.2026.1708003 (PMC12975138; doi:10.3389/fspor.2026.1708003)
Supplement: Supplementary file 1 [file Datasheet1.pdf]

JBI Cohort Studies Risk of Bias Template

Author (Year)	Q1. Two groups similar and recruited from same population	Q2. Exposures measured similarly to assign exposure status	Q3. Exposure measured validly and reliably	Q4. Confounding factors identified	Q5. Strategies to deal with confounding stated	Q6. Participants free of outcome at start of study	Q7. Outcomes measured validly and reliably	Q8. Follow-up time reported and sufficient	Q9. Complete follow-up or reasons described	Q10. Strategies to address incomplete follow-up	Q11. Appropriate statistical analysis used	Overall Appraisal (High / Moderate / Low)
Yates et al. (2020)	Yes	Yes	Yes	Yes	Yes	Yes	Yes	Yes	No	No	Yes	High(9)
Yerramalla et al. (2021)	Yes	Yes	Yes	Yes	Yes	Yes	Yes	Yes	No	No	Yes	High(9)
Peter-Marske et al. (2023)	Yes	Yes	Yes	Yes	Yes	Yes	Yes	Yes	No	No	Yes	High(9)
